# Maternal Adiposity and DNA Methylation of TfR2 and HJV Genes in Early Pregnancy: Mediating Role of Inflammation and Consequences for Iron Status

**DOI:** 10.1016/j.tjnut.2026.101393

**Published:** 2026-02-04

**Authors:** Sabrina P Demirdjian, Rachelle E Irwin, Paul D Thompson, Maria S Mulhern, Maeve A Kerr, Mark Ledwidge, Kazi L Rahman, Caroline Conway, Edna P Rodriguez, Mary T McCann

**Affiliations:** 1 Nutrition Innovation Centre for Food and Health, School of Biomedical Sciences, Ulster University, Coleraine, United Kingdom; 2 School of Biomedical Sciences, Biomedical Sciences Research Institute, Ulster University, Coleraine, United Kingdom; 3 School of Medicine, University College Dublin, Dublin, Ireland

**Keywords:** cytosine-phosphate-guanine, CpG islands, hemojuvelin, transferrin receptor, epigenomics, iron deficiencies, gravidity, overweight, interleukin, cytokine

## Abstract

**Background:**

Growing evidence shows that obesity influences iron status during pregnancy; however, it is unknown whether maternal obesity is associated with epigenetic changes in transferrin receptor 2 (*TfR2)* and hemojuvelin (*HJV)*.

**Objectives:**

This study aimed to explore the association between adiposity and DNA methylation in *TfR2* and *HJV* in early pregnancy and the mediating effect of inflammation on this association.

**Methods:**

This cross-sectional study used data from a double-blind randomized controlled trial in singleton pregnant women with normal weight (BMI: 18.5–24.9 kg/m^2^) and obesity (BMI: ≥30.0 kg/m^2^). Maternal BMI, fat mass, visceral fat, iron/inflammatory markers and DNA methylation of *TfR2* and *HJV* were measured at 12 gestational weeks. Two primer sets were designed [*TfR2 zone 1 and 2* (*TfR2z1*, *TfR2z2*); *HJV zone 1 and 2* (*HJVz1, HJVz2*)]. An inflammation score was calculated using proinflammatory cytokines.

**Results:**

A total of 65 pregnant women were included, 34 with normal weight, and 31 with obesity. Compared to women of normal-weight, those with obesity showed: lower percentage DNA methylationat *TfR2z1* cytosine-phosphate-guanine (CpG) sites 5, 6, 8–10 and the mean *TfR2z1* methylation (mean *TfR2z1* methylation 5.80% vs 6.92%, *P =* 0.004); lower percentage of DNA methylation at *TfR2z2* CpG sites 5 and 6 (12.5% vs 14.7%, *P =* 0.035; 17.8% vs 20.1%, *P =* 0.031); higher percentage DNA methylation at *HJVz1* CpG site 3 (*HJVz1* CpG 3 45.3% vs 43.4%, *P =* 0.010) and *HJVz2* CpG site 2 and the mean *HJVz2* methylation (*HJVz2* CpG 2 43.2% vs 37.9%, *P <* 0.001, mean *HJVz2* methylation 65.9% vs 61.6%, *P <* 0.001). Adjusting for covariates, DNA methylation at *TfR2z1* was negatively and *HJVz2* positively associated with all adiposity measures (*TfR2z1*, BMI *β* = –0.288, *P* = 0.030; *HJVz2 β* = 0.459, *P <* 0.001). Inflammation showed a mediating effect on the association between all adiposity measures and DNA methylation at *HJVz1* (*P* = 0.019).

**Conclusions:**

Maternal adiposity is associated with epigenetic alterations in the iron metabolism genes *TfR2* and *HJV* in early pregnancy, with modifications in *HJV* appearing to be mediated by inflammatory pathways.

## Introduction

Obesity and iron deficiency (ID) are major pregnancy risks, both having high global prevalence and associated with severe health complications [1,2]. Obesity rates continue to rise, particularly in high-income regions [3], whereas ID remains widespread, affecting up to 80% of pregnant women in low-income countries and 45% in high-income settings [4]. Evidence increasingly links obesity to impaired iron metabolism. Although findings remain inconsistent, studies show that pregnant women with obesity often exhibit compromised iron status, with elevated soluble transferrin receptor (sTfR) in early and mid-pregnancy [[5], [6], [7], [8], [9], [10]], and reduced serum iron [7,[11], [12], [13], [14], [15]], compared to women without obesity. Iron deficiency anemia appears more common in women with central obesity [16], likely driven by chronic low-grade inflammation, which may upregulate hepcidin and disrupt iron homeostasis [[17], [18], [19]]. Despite the high prevalence of maternal obesity and ID, the underlying mechanisms driving these alterations remain poorly understood.

DNA methylation is a key regulatory mechanism of gene expression and has been identified in genes involved in iron metabolism. This regulatory pathway is catalyzed by methyltransferases that transfer a methyl group from S-adenosyl methionine to the fifth carbon of cytosine at cytosine-phosphate-guanine (CpG) sites, forming 5-methylcytosine (5 mC) [20]. DNA segments having a higher number of CpG sites are known as CpG islands, usually unmethylated [21], and are frequently found in gene promoters. Methylation of CpG islands usually leads to stable silencing of the gene [22]. DNA methylation patterns vary by tissue type; however, CpG islands do not usually exhibit these tissue-specific methylation patterns [23]. In vitro and animal studies show that ID causes epigenomic changes in iron regulatory genes such as *HAMP, DMT1, FPN,* and *Dcytb* [24]. Despite not yet being replicated in human studies, these data suggest that iron status may influence the epigenetic regulation of these genes.

Hemojuvelin (HJV) is a membrane protein that senses the concentrations of iron in the portal circulation, derived from iron absorbed from the diet in the intestine, and promotes hepcidin synthesis to prevent iron overload [25]. HJV expression rises during iron overload, likely promoting hepcidin production as a compensatory mechanism to restore iron balance [26]. However, it is unknown how iron status and *HJV* DNA methylation, in the context of inflammation and obesity, modulate its expression.

Transferrin receptor 2 (TfR2) is a liver membrane receptor for transferrin, the protein responsible for iron transport in the bloodstream. Iron-bound transferrin is mainly internalized via transferrin receptor 1 (TfR1), whereas TfR2 plays a broader regulatory role in iron metabolism [27]. Mice lacking TfR1 do not survive, whereas mice lacking TfR2 survive but develop severe iron overload, indicating that TfR2 complements TfR1 in regulating iron homeostasis [28]. In the liver, TfR2 forms a complex with HJV to control hepcidin synthesis. In the bone marrow, TfR2 associates with the erythropoietin (EPO) receptor to support red blood cell production, particularly when iron is scarce [27,28].

*HJV* and *TfR2* are key regulators of iron metabolism, and both may be influenced by inflammation. Tumor Necrosis Factor (TNF)-α is suggested to downregulate *HJV* expression in animal models [29], shifting hepcidin regulation from iron status to inflammation [30,31]. Under normal conditions, high transferrin saturation (TSAT) increases *TfR2* expression, promoting hepcidin production to reduce iron concentrations. Wallace et al. [32] report that inflammation-induced hepcidin was weaker in *TfR2*-deficient mice, suggesting that inflammation mediates this response. TNF-α and interleukin (IL)-6 both increase *TfR2* expression, though IL-6 requires additional serum factors [33]. In summary, inflammation may override iron-based regulation by suppressing *HJV* and enhancing *TfR2*, making hepcidin production driven by inflammation rather than iron concentrations, which may disrupt iron balance.

Obesity and suboptimal iron status in early pregnancy have the potential to severely impact maternal and fetal health. Understanding pathways linking obesity, inflammation, and iron metabolism is crucial for identifying women at risk and guiding targeted interventions and policies.

The primary objective of this study was to explore the association between maternal adiposity and DNA methylation of *TfR2* and *HJV* genes in early pregnancy. As secondary objectives, we examined the association between DNA methylation in *TfR2* and *HJV* with iron/inflammatory markers and explored the potential role of inflammatory markers as mediators of changes in DNA methylation in women with high adiposity.

## Methods

### Study design

This was a cross-sectional study using data from a double-blinded randomized controlled intervention study [MO-VITD trial]. The original study details and its findings are reported by Alhomaid et al. [34] (15/NI/0068, WHSCT reference number 14/49).

### Study participants

The MO-VITD trial recruited healthy singleton pregnancies in the first trimester without pregnancy-related complications, aged ≥18 y, and with BMI ≥18.5 kg/m^2^. After completion of informed consent, a blood sample was taken and body composition and maternal anthropometric measurements by bioelectrical impedance (TANITA MC-780MA) were recorded. Participants were randomly assigned to receive either 10 or 20 μg/d of vitamin D_3_ from 12 gestational weeks (GW) through to delivery. A total of 240 participants were recruited; 120 participants received a multivitamin plus 10 μg/d vitamin D, and 120 participants received a multivitamin plus 20 μg/d vitamin D. The multivitamin contained 17 mg of elemental iron. At 12, 28, and 36 GW, blood samples were collected, and an umbilical cord blood sample was taken at delivery. All participants completed a health and lifestyle questionnaire.

For this analysis, baseline data (12 GW) from participants who consented to the use of their data for future studies were utilized. Pregnant women with diabetes mellitus, chronic liver or renal disease, autoimmune disorders, acute infections, chorioamnionitis, hematological disorders, or malabsorptive syndromes were excluded.

### Data source

Data already collected in the MO-VITD study were used: maternal anthropometry, body composition at 12 GW, maternal C-reactive protein (CRP) concentrations, obstetric, and neonatal clinical data.

Hematological markers, including hemoglobin, red cell blood count (RBC), mean cell volume, mean cell hemoglobin, red cell distribution width, and mean cell hemoglobin concentration from 12 GW, were collected from maternal medical notes. Ethical approval was obtained (23/NI/0087, REC reference number 321833, research governance number 23/0033) to access maternal medical notes.

### Biochemical analyses

Measurement of iron and inflammatory markers was performed with stored serum samples, which were taken at 12 GW, immediately before commencement of trial supplementation. The iron markers measured were: serum ferritin, transferrin, serum iron, hepcidin, sTfR; and the inflammatory markers were IL-6, TNF-α, IL-1β, and interferon-γ (IFN-γ). We prioritized the measurement of short-lived proinflammatory cytokines as these markers are integral to the chronic inflammatory phenotype associated with obesity and have consistently been reported as elevated in women with obesity and central adiposity. Furthermore, proinflammatory cytokines are considered key biomarkers of chronic low-grade inflammation, making them relevant for understanding chronic disease processes [35,36]. TSAT, unsaturated iron binding capacity (UIBC), and total iron binding capacity (TIBC) were calculated from transferrin and serum iron measurements [UIBC = TIBC-serum iron and TSAT = ($\frac{serum\;iron}{TIBC}$) × 100] [37]. Ferritin, serum iron and transferrin analyses were performed at Causeway Hospital laboratory, Coleraine, Northern Ireland, using Elecsys Ferritin, cobas, Roche; IRON2, cobas, Roche; and TRSF2 Tina-quant Transferrin ver.2, cobas, Roche, respectively. Hepcidin, sTfR, and inflammatory markers (IFN-γ, IL-1β, IL-6, and TNF-α) analyses were performed at the Nutrition Innovation Centre for Food and Health laboratory, Coleraine, Ulster University, using Quantikine ELISA, Human Hepcidin Immunoassay, R&D Systems; Human sTfR ELISA BioVendor Group; and S-PLEX Platform, Proinflammatory Panel 1 (human) Kit, MSD, respectively. Ferritin was adjusted for inflammation using CRP concentrations applying internal regression correction [38,39].

The ELISA kit used for sTfR analysis provides a reference range derived from data on 153 adults (89 men and 69 women) aged 18–43 y. Reported descriptive statistics include a mean of 0.868 mg/L, median of 0.854 mg/L, minimum of 0.072 mg/L, maximum of 1.699 mg/L, SD of 0.307 mg/L, and SEM of 0.024 mg/L. The reference interval, corresponding to the 2.5th and 97.5th percentiles, spans 0.378–1.513 mg/L.

### DNA methylation analysis

#### Primer selection

For the *TfR2* gene, primers were designed based on sequence within the promoter/enhancer region located on chr7:100,641,057–100,641,789; and in the CpG island “CpG 57” located on chr7:100,633,027–100,633,697. For the *HJV* gene, primers were designed based on the promoter region located on chr1:146,021,170–146,022,009; and on CpG island “CpG 29” located on chr1:146,019,305–146,019,601. Supplementary Tables 1 and 2 provide the characteristics of each primer used in this analysis [*transferrin receptor* *2* *zone 1 (TfR2z1); transferrin receptor* *2* *zone 2 (TfR2z2), hemojuvelin zone 1* (*HJVz1), hemojuvelin zone 2* (*HJVz2*]. For the alignment of genomic sequences, BLAST-Like Alignment Tool) was used to find sequences in the GRCh38/hg38 genome assembly that have ≥95% similarity over an extension of ≥25 bases, using the University California Santa Cruz Genome Browser. Regarding *TfR2* isoforms, the full-length gene corresponds to the α isoform. Both primers target this α isoform; however, only sites 1 and 2 within *TfR2z1* are common to both isoforms. Primer design was performed in-house using PyroMark Assay Design version 2.0.1.15 2008 QIAGEN Group.

#### DNA methylation analysis

DNA methylation analysis was conducted using stored maternal buffy coat samples obtained at 12 GW and collected before the start of the vitamin D and multivitamin supplementation. The samples were stored at −80°C from the time of collection in the original study and have not been used since. DNA extraction was performed using the PerkinElmer DNA extraction machine (2024-0020), Chemagic 360. The concentration and purity of genomic DNA in each sample were determined using Nanodrop 2000 Spectrophotometer. Purified genomic DNA was bisulfite converted using the EZ DNA Methylation-Lightning TM Kit according to manufacturers’ instructions (Zymo Research). Bisulfite converted DNA was amplified using designed primers via pyro-PCR and conducted using the PyroMarkTM PCR kit (Qiagen) according to manufacturer’s instructions. The annealing temperatures used for each primer set were: *TfR2z1* 55.5°C, *TfR2z2* 56.3°C, *HJVz1*: 53°C, and *HJVz2* 56°C. Pyrosequencing was conducted on the PyroMark Q48 instrument.

### Variables and definitions

BMI, fat mass, visceral fat, and fat mass index [fat mass in kg/(height in m)^2^] (FMI) were considered independent variables. Pregnant women were categorized as either normal weight (BMI: 18.5–24.9 kg/m^2^) or with obesity (BMI: ≥30 kg/m^2^) [40]; and according to body fat categories of low body fat (<35%) or high body fat (≥35%) [41].

Hematological and iron markers were considered independent variables and inflammatory markers as mediators. ID was defined as serum ferritin <30 μg/L [42]. Percentage of DNA methylation in the selected *TfR2* and *HJV* zones was the outcome.

A modified version of an inflammation score published elsewhere [43] was used to categorize the sample population into high and low inflammation dependent on the concentrations of proinflammatory cytokines measured at baseline. Cytokines (IL-6, TNF-α, IFN-γ, and IL-1β) were split into tertiles and then allocated a score with 1 point to tertile 1, 2 points to tertile 2, and 3 points to tertile 3, which were then summed to obtain the inflammation score. The inflammation score was categorized as either high or low inflammation, according to scores above or below the 50th centile (high inflammation score ≥8, low inflammation score <8). Low and high inflammation were also defined using CRP concentrations ≤5 and >5 mg/L [44]. Age, smoking status, educational level, and hemoglobin concentration at 12 GW were considered covariates.

### Statistical methods

Statistical analyses were performed using SPSS (Statistical Package for the Social Sciences software, version 29; IBM). Normality was checked using Kolmogorov–Smirnov test with the results presented as mean ± SD or absolute frequency and percentage, as appropriate. Mean methylation percentages of each zone of the *TfR2* and *HJV* genes were compared between the normal weight and obesity groups, between low and high body fat %, between high and low inflammation groups, and between those with ferritin <30 and ≥30 μg/L using general linear models, adjusting for covariates.

Pearson correlation was used to explore the relationship between hematological, iron and inflammatory markers and the DNA methylation percentage of each of the evaluated zones of *TfR2* and *HJV*.

Multiple linear regression analysis was used to evaluate the association between DNA methylation percentage in each CpG site and adiposity. The inflammation score was considered a mediator, generating the following interaction terms: BMI∗inflammation, fat mass∗inflammation, visceral fat∗inflammation, and FMI∗inflammation. We also investigated the independent association between CRP and the inflammation score with the mean methylation percentage in each assessed region of these genes. Linear regression analysis was adjusted for covariates as described. *P* < 0.05 was considered significant.

## Results

**FIGURE 1:**
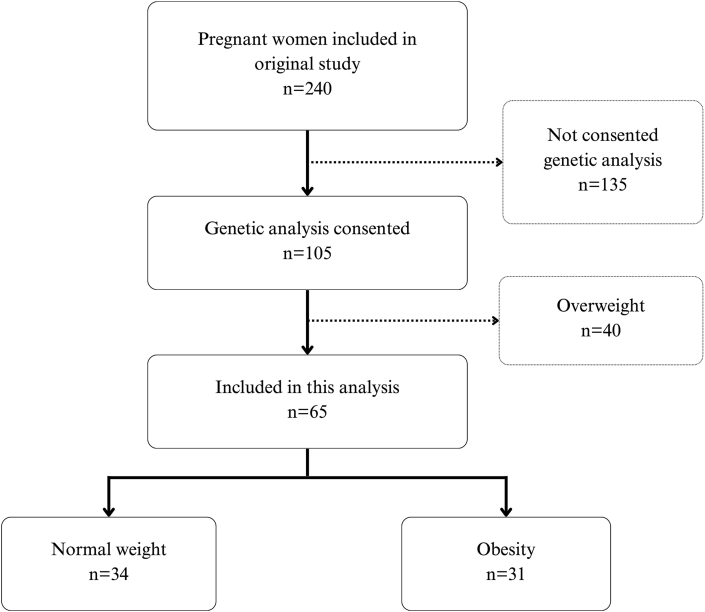
Flowchart showing the selection process of the included participants.

Of the 240 participants included in the original MO-VITD study, 105 agreed to have their samples used for genetic studies. Of these, 34 were of normal weight, 40 were overweight, and 31 had obesity. For this analysis, only pregnant women with normal weight and obesity were included (*n* = 65) (Figure 1). Table 1 describes the characteristics of the population, showing that the obesity group had lower educational level (*P =* 0.040), and higher systolic and diastolic blood pressure at 12 GW (*P <* 0.001) compared to normal-weight group. The mean concentrations of each iron and inflammatory marker in both groups (normal weight and obesity) are shown in Supplementary Table 3. Compared to women with normal weight, those with obesity had higher hemoglobin concentrations (132 vs 126 g/L, *P =* 0.017), lower serum iron and TSAT (15.7 vs 19.6 μmol/L, *P =* 0.009; 23.8% vs 29.9%, *P =* 0.011, respectively) and higher CRP and IL-6 (CRP 11.7 vs 4.44 mg/L, *P <* 0.001; IL-6: 2231 vs 1423 fg/mL, *P =* 0.002). Pregnant women with CRP >5 mg/L had higher IL-6 and IL-1β compared to those with CRP ≤5 mg/L (IL-6: 2235 vs 1436 fg/mL, *P <* 0.001; IL-1β 179 vs 126 fg/mL, *P =* 0.039). No significant differences in iron or inflammatory markers were observed between women who received iron supplements before 12 GW and those who did not, in either BMI group. The only exception was a higher hepcidin concentration among normal-weight women taking iron-containing supplements (median 9997 vs 13517 pg/mL; *P =* 0.016).

**TABLE 1:** General characteristics of MO-VITD participants included in this analysis.

	Normal-weight group (n = 34)				Obesity *n =* 31				*P* value
	Mean	SD	*n*	%	Mean	SD	*n*	%	
Age (y)	30.6	5.1	—	—	29.6	4.9	—	—	0.410
Weight (kg)	60.5	6.4	—	—	92.5	14	—	—	<0.001
Height (m)	1.63	0.06	—	—	1.64	0.05	—	—	0.823
Body fat (%)	27.4	4.9	—	—	41.0	4.0	—	—	<0.001
Visceral fat (cm^2^)	2.6	1.0	—	—	8.2	2.3	—	—	<0.001
Fat mass index (kg/m^2^)	6.26	1.4	—	—	14.2	3.0	—	—	<0.001
Gestational age (wk)	13.1	1.3	—	—	12.8	1.3	—	—	0.363
Systolic BP (mmHg)	115	11.7	—	—	124.8	10.1	—	—	<0.001
Diastolic BP (mmHg)	69.3	9.6	—	—	73.3	7.5	—	—	<0.001
Education at 3rd level or above, *n* (%)	—	—	26	76.5	—	—	14	51.7	0.040
Smoking, *n* (%)	—	—	2	5.9	—	—	3	10	0.540
Parity, *n* (%)									
1	—	—	10	30.3	—	—	13	43.3	0.557
2	—	—	13	39.4	—	—	10	33.3	—
≥3	—	—	10	30.3	—	—	7	23.3	—
Previous miscarriage, *n* (%)	—	—	11	32.4	—	—	8	25.8	0.562
Iron supplementation, *n* (%)	—	—	17	51.5	—	—	9	29.0	0.067
Iron deficiency,^1^*n* (%)	—	—	10	29.4	—	—	5	16.1	0.204
Iron deficiency (sTfR),^2^*n* (%)	—	—	3	8.8	—	—	7	23.3	0.111
Mode of delivery									
Vaginal, *n* (%)	—	—	19	57.6	—	—	16	53.3	0.131
Elective C-section, *n* (%)	—	—	6	18.2	—	—	4	13.3	—
Emergency C-section, *n* (%)	—	—	4	12.1	—	—	10	33.3	—
Blood loss (mL)	446.9	264.8	—	—	556.3	367.7	—	—	0.179
GA delivery (wk)	39.6	1.8	—	—	39.9	1.4	—	—	0.502
Childbirth characteristics									
Birth weight (g)	3580.9	590.5	—	—	3633.1	420.4	—	—	0.731
Length (cm)	52.3	4.8	—	—	53.3	2.9	—	—	0.373
Head circumference (cm)	36.0	4.1	—	—	35.0	1.0	—	—	0.278
Apgar 1 min	8.5	0.9	—	—	8.4	1.0	—	—	0.768
Apgar 5 min	8.9	0.6	—	—	8.9	0.4	—	—	0.872
Gender female, *n* (%)	—	—	13	50	—	—	15	68.2	0.203

Data presented as mean ± SD or absolute number and percentage. Independent Student’s t-test used for continuous and χ^2^ for categorical variables. The sample size varies across certain variables: Smoking: normal weight (*n =* 34), obesity (*n =* 30); parity: normal weight (*n =* 33), obesity (*n =* 30), education: normal weight (*n =* 34), obesity (*n =* 29); mode of delivery: normal weight (*n =* 33), obesity (*n =* 30); forceps/vacuum deliveries are not shown due to low frequency. Normal weight BMI: 18.5–24.9 kg/m^2^, obesity BMI: ≥30 kg/m^2^.

Abbreviations: BP, blood pressure; C-section, cesarean section; sTfR, soluble transferrin receptor.

1 Ferritin <30 μg/L.

2 sTfR>1.513 μg/mL.

**FIGURE 2:**
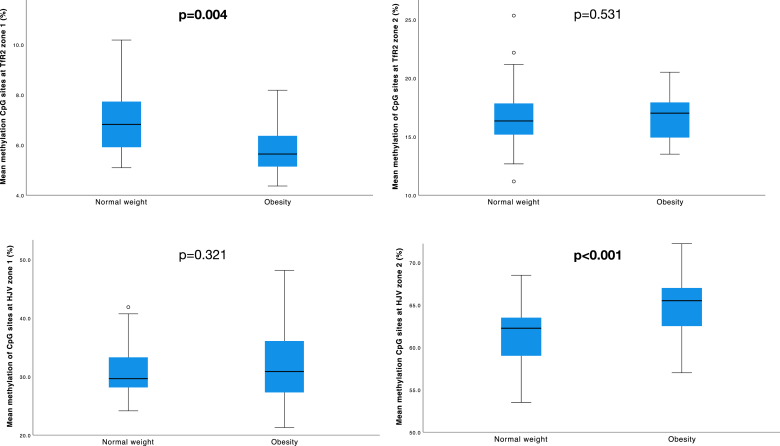
Box plots showing the distribution of the percentage DNA methylation for each evaluated zone in the *TfR2* and *HJV* genes. Data shown as percentage methylation median, IQR, 25 and 75 percentiles. General linear model adjusted for maternal age, smoking status, educational status, and hemoglobin at baseline. *P* < 0.05 considered significant. Normal weight: BMI 18.5–24.9 kg/m^2^, obesity: BMI ≥30 kg/m^2^. CpG, cytosine-phosphate-guanine; HJV, hemojuvelin; TfR2, transferrin receptor 2.

The mean percentage DNA methylation across all CpG sites of *TfR2* (zones 1 and 2) was lower in women with obesity compared to those with normal weight (9.6% vs 10.4%, *P =* 0.025). The mean percentage of overall DNA methylation for each gene zone evaluated was: *TfR2z1* 6.39% ± 1.2%, *TfR2z2* 16.7% ± 2.4%*, HJVz1* 31.4% ± 5.3%, *HJVz2* 63.2% ± 3.9%. Table 2 shows that in *TfR2*z1, the mean DNA methylation at several CpG sites was lower in women with obesity compared to those with normal weight (mean of all CpG sites 5.80% vs 6.92%, *P =* 0.004; CpG 5 6.22% vs 8.06%, *P <* 0.001, CpG 6 9.84% vs 12.4%, *P <* 0.001, CpG 8 4.80% vs 6.11%, *P =* 0.006, CpG 9 5.77% vs 6.94%, *P =* 0.008, and CpG 10 9.54% vs 12.6%, *P <* 0.001, in obesity and normal weight, respectively) (Figure 2). At *TfR2z2*, DNA methylation at CpG sites 5 and 6 were lower in women with obesity compared to those with normal weight (12.5% vs 14.7%, *P =* 0.035; 17.8% vs 20.1%, *P =* 0.031).

**TABLE 2:** DNA methylation (%) of *TfR2* and *HJV* genes at each CpG site according to BMI group.

	Normal weight		Obesity		*P* value
	Mean	SD	Mean	SD	
*TfR2*z1 (%)					
CpG 1	3.82	1.24	3.54	0.76	0.864
CpG 2	5.08	1.91	4.74	1.45	0.814
CpG 3	5.79	1.36	5.45	1.23	0.972
CpG 4	4.41	1.13	4.00	0.85	0.411
CpG 5	8.06	1.41	6.22	1.14	<0.001
CpG 6	12.4	2.54	9.84	1.91	<0.001
CpG 7	4.61	1.01	4.48	1.18	0.919
CpG 8	6.11	2.07	4.80	1.3	0.006
CpG 9	6.94	2.03	5.77	1.28	0.008
CpG 10	12.6	2.26	9.54	1.33	<0.001
CpG 11	6.20	1.41	5.48	1.54	0.339
Mean of all CpG sites	6.92	1.27	5.80	0.96	0.004
*TfR2*z2 (%)					
CpG 1	14.8	4.16	16.1	4.28	0.180
CpG 2	13.4	2.85	15.1	2.27	0.103
CpG 3	26.3	6.23	28.2	3.98	0.103
CpG 4	11.7	6.91	9.9	1.77	0.100
CpG 5	14.7	4.73	12.5	2.08	0.035
CpG 6	20.1	5.58	17.8	2.82	0.031
Mean of all CpG sites	16.8	2.75	16.6	2.03	0.531
*HJV*z1 (%)					
CpG 1	49.3	5.12	50.0	6.43	0.382
CpG 2	52.3	7.99	51.2	9.57	0.822
CpG 3	43.4	4.46	45.3	5.21	0.010
CpG 4	26.8	7.22	26.7	9.87	0.807
CpG 5	13.3	4.05	14.0	5.00	0.372
CpG 6	17.0	6.35	18.3	7.94	0.332
CpG 7	15.5	6.91	16.6	9.14	0.266
Mean of all CpG sites	31.1	4.47	31.7	6.23	0.321
*HJV*z2 (%)					
CpG 1	85.4	5.43	86.7	2.62	0.084
CpG 2	37.9	3.18	43.2	6.59	<0.001
Mean of all CpG sites	61.6	3.32	65.0	3.96	<0.001

Data presented as mean % methylation ±SD. "Mean of all CpG sites" refers to the mean percentage of DNA methylation across all CpG sites within the zone being assessed. General linear model adjusted for maternal age, smoking, educational status, and hemoglobin at 12 gestational weeks. BMI: 18.5–24.9 kg/m^2^, obesity BMI: ≥30 kg/m^2^.

Abbreviations: CpG, cytosine and guanine separated by a single phosphate group; HJV, hemojuvelin; HJVz1, hemojuvelin zone 1; HJVz2, hemojuvelin zone 2; TfR2, transferrin receptor 2; TfR2z1, transferrin receptor 2 zone 1; TfR2z2, transferrin receptor 2 zone 2.

The percentage DNA methylation of all *HJV* CpG sites between both BMI categories was similar (*P =* 0.083). At *HJV*z1, only CpG 3 was higher in the obesity group (45.3% vs 43.4%, *P =* 0.010), with no other observed differences. At *HJV*z2, higher DNA methylation was observed in the obesity group at CpG site 2 and the mean methylation of the 2 CpG sites (43.2% vs 37.9%, *P <* 0.001; 65.0% vs 61.6%, *P <* 0.001, respectively) (Table 2 and Figure 2). Comparison of percentage DNA methylation between low and high body fat pregnant women showed similar results.

When comparing methylation percentages between women with CRP concentrations >5 mg/L and those with ≤5 mg/L, lower methylation was observed at *TfR2*z1 CpG sites 5, 6, 10 and the mean of the zone (6.69% vs 7.78%, *P =* 0.012; 9.80% vs 12.5%, *P <* 0.001, 10.0% vs 12.3%, *P <* 0.001, and 6.01% vs 6.82%, *P =* 0.030, respectively), as well as at *TfR2*z2 CpG sites 5 and 6, and in the mean DNA methylation percentage of the zone (11.9% vs 15.2%, *P =* 0.001; 17.2% vs 20.5%, *P =* 0.008; 15.9% vs 17.2%, *P =* 0.029, respectively) (Supplementary Table 4).

No differences were found in methylation percentages at any of the CpG sites or in the mean percentage DNA methylation across the regions studied between women with and without ID, whether defined as ferritin <30 μg/L or sTfR >1.513 mg/L. No differences in DNA methylation of any evaluated genewere observed between women who received iron supplementation before 12 GW and those who did not, regardless of BMI category.

In linear regression analysis, adjusting for maternal age, educational level, smoking and hemoglobin at 12 GW, all adiposity measures (BMI, fat mass, visceral fat, and FMI) were negatively associated with the mean percentage methylation at *TfR2z1* (particularly fat mass *β =* –0.331, *P =* 0.011; visceral fat *β =* –0.304, *P =* 0.022; and FMI *β =* –0.333, *P =* 0.011) and positively with *HJVz2* (particularly BMI: *β =* 0.459, *P <* 0.001; visceral fat *β =* 0.447, *P <* 0.001; and FMI *β =* 0.418, *P =* 0.001) (Table 3). Inflammation score was not independently associated with methylation in any of the zones evaluated in both genes. However, the presence of inflammation, measured with the inflammation score ≥8, showed a mediating effect in the association between adiposity and the percentage DNA methylation of *HJV*z1 (Table 3), interpreting that as adiposity increases in the presence of high inflammation, methylation in *HJV*z1 decreases.

**TABLE 3:** Association between DNA methylation of *TfR2* and *HJV* and adiposity measures in early pregnancy, considering the inflammation score as a potential mediator.

Mean DNA methylation (%) of all CpG sites at each zone	BMI (kg/m^2^)	BMI∗IS	Fat mass (%)	Fat mass∗ IS	Visceral fat	Visceral fat∗IS	FMI kg/m^2^	FMI∗IS
*TfR2*z1								
β	–0.288	–0.192	–0.331	0.046	–0.304	0	–0.333	–0.112
B	–0.056	–0.003	–0.033	0	–0.122	0	–0.094	–0.003
CI 95%	–0.107, –0.005	–0.03,0.03	–0.06,–0.01	–0.01, 0.01	–0.225, –0.018	–0.06, 0.06	–0.16,–0.02	–0.04, 0.04
P value	0.030	0.856	0.011	0.954	0.022	1.00	0.011	0.893
*TfR2*z2								
β	–0.112	–1.490	–0.078	–1.232	–0.090	–1.296	–0.098	–1.296
B	–0.043	–0.043	–0.016	–0.023	–0.071	–0.102	–0.055	–0.067
CI 95%	–0.15, 0.06	–0.10, 0.02	–0.07, 0.04	–0.05, 0.01	–0.29, 0.15	–0.23, 0.03	–0.21, 0.10	–0.16, 0.02
P value	0.429	0.191	0.574	0.160	0.525	0.133	0.486	0.153
*HJV*z1								
β	0.070	–2.554	0.077	–2.000	0.074	–1.825	0.114	–1.932
B	0.058	–0.155	0.033	–0.079	0.125	–0.305	0.136	–0.214
CI 95%	–0.16, 0.28	–0.28,–0.02	–0.08, 0.14	–0.14, –0.01	–0.33, 0.58	–0.57, –0.03	–0.18, 0.46	–0.40,–0.02
P value	0.607	0.019	0.567	0.016	0.589	0.027	0.403	0.025
*HJV*z2								
β	0.459	–0.263	0.388	–0.155	0.447	–0.200	0.418	–0.100
B	0.285	–0.012	0.125	–0.005	0.569	–0.025	0.376	–0.008
CI 95%	0.13, 0.43	–0.10, 0.08	0.04, 0.20	–0.05, 0.04	0.25, 0.88	–0.22, 0.20	0.15, 0.60	–0.14, 0.13
P value	<0.001	0.792	0.003	0.845	<0.001	0.793	0.001	0.902

Data presented as *β* coefficient, unstandardized B, and 95% CI. "Mean DNA methylation of all CpG sites of each zone" refers to the mean percentage of DNA methylation across all CpG sites within the zone being assessed. Multiple linear regression, adjusted for age, smoking, educational level, and hemoglobin concentrations.

Abbreviations: CI, confidence interval; CpG, cytosine and guanine separated by a single phosphate group; FMI, fat mass index; HJV, hemojuvelin; HJVz1, hemojuvelin zone 1; HJVz2, hemojuvelin zone 2. IS, inflammation score; TfR2, transferrin receptor 2; TfR2z1, transferrin receptor 2 zone 1; TfR2z2, transferrin receptor 2 zone 2.

When the relationship between iron markers and DNA methylation of these zones was analyzed, we found that RBC correlated negatively with the percentage DNA methylation in *TfR2*z1 CpG 2, 6 and 10 (*P <* 0.050), and sTfR correlated positively with CpG site 2 of *TfR2*z2 (*P <* 0.001). TSAT correlated negatively with percentage DNA methylation of *HJV*z1 CpG 3, 4, 5, 7, and the mean methylation of the zone, and serum iron also correlated negatively with CpG 4, 5, 6, 7, and the mean methylation of this zone (TSAT *r =* –0.279, *r =* –0.283, *r =* –0.290, *r =* –0.295, *r =* –0.284, serum iron *r =* –0.270, *r =* –0.307, *r =* –0.264, *r =* –0.302, *r =* –0.273; all *P <* 0.050, respectively) (Table 4). Likewise, UIBC correlated positively with methylation of *HVJz1* at CpG sites 3, 4, 7, and the mean percentage methylation of this zone (*r =* 0.299, *r =* 0.291, *r =* 0.248, *r =* 0.267; *P <* 0.050, respectively), so this zone correlated inversely with an ID status. Hemoglobin correlated positively with percentage DNA methylation in *HJV*z2, CpG site 2 and the mean percentage methylation (*r =* 0.361, *P <* 0.010; *r =* 0.281, *P <* 0.050) and RBC positively correlated with *HJV*z2 CpG site 2 (*r =* 0.323, *P <* 0.050). The mean percentage methylation of both zones of *TfR2* correlated negatively with hepcidin (*r =* –0.247, *P =* 0.057) and the mean of both zones of *HJV* correlated positively with UIBC (*r =* 0.269, *P =* 0.030), and negatively with TSAT (*r =* –0.286, *P =* 0.021), and serum iron (*r =* –0.272, *P =* 0.028).

**TABLE 4:** Relationship between iron markers and percentage methylation of *TfR2* and *HJV*.

	Hb (g/L)	RBC (10^12^/L)	UIBC (μmol/L)	TSAT (%)	Fe (μmol/L)	sTfR (μg/mL)
*TfR2*z1 (%)						
CpG 1	–0.205	–0.238	–0.020	–0.054	–0.071	0.011
CpG 2	–0.217	–0.277∗	0.014	–0.051	–0.044	–0.086
CpG 3	–0.106	–0.098	0.096	–0.121	–0.064	0.058
CpG 4	–0.174	–0.168	0.143	–0.125	–0.068	0.005
CpG 5	–0.150	–0.227	0.029	0.121	0.241	0.009
CpG 6	–0.128	–0.288∗	–0.112	0.165	0.219	–0.085
CpG 7	–0.148	–0.072	0.177	–0.112	–0.037	0.073
CpG 8	0.021	–0.039	–0.013	0.109	0.176	–0.072
CpG 9	0.062	–0.019	0.035	0.061	0.141	–0.065
CpG 10	–0.184	–0.299∗	–0.070	0.145	0.227	–0.070
CpG 11	–0.223	–0.256	–0.071	0.161	0.216	0.092
Mean of all CpG sites	–0.164	–0.247	–0.002	0.071	0.149	–0.033
*TfR2*z2 (%)						
CpG 1	–0.091	0	0.049	–0.052	–0.043	0.186
CpG 2	0.164	0.147	0.179	–0.071	0.017	0.412∗∗∗
CpG 3	–0.009	–0.095	–0.051	0.099	0.123	0.005
CpG 4	0.126	0.130	0.088	–0.053	0.007	0.195
CpG 5	–0.001	0.094	0.041	–0.028	0.010	0.009
CpG 6	–0.004	0.024	–0.031	0.042	0.063	–0.073
Mean of all CpG sites	0.044	0.072	0.062	–0.005	0.061	0.182
*HJV*z1 (%)						
CpG 1	–0.182	–0.224	0.104	–0.048	0.005	0.058
CpG 2	–0.051	–0.007	0.142	–0.139	–0.137	–0.090
CpG 3	–0.152	–0.104	0.299∗	–0.279∗	–0.223	–0.059
CpG 4	–0.241	–0.153	0.291∗	–0.283∗	–0.270∗	–0.029
CpG 5	–0.227	–0.154	0.223	–0.290∗	–0.307∗	–0.003
CpG 6	–0.148	–0.115	0.173	–0.240	–0.264∗	–0.083
CpG 7	–0.211	–0.118	0.248∗	–0.295∗	–0.302∗	–0.099
Mean of all CpG sites	–0.214	–0.154	0.267∗	–0.284∗	–0.273∗	–0.064
*HJV*z2 (%)						
CpG 1	0.033	–0.079	–0.091	0.100	0.104	–0.185
CpG 2	0.361∗∗	0.323∗	0.070	–0.073	–0.056	0.152
Mean of all CpG sites	0.281∗	0.190	0.001	0.002	0.016	0.008

Pearson linear correlation. Data presented as coefficient *r*.

Only markers that had a significant correlation are shown."Mean of all CpG sites" refers to the mean percentage of DNA methylation across all CpG sites within the zone being assessed.

Abbreviations: CpG, cytosine and guanine separated by a single phosphate group; Fe, serum iron; Hb, hemoglobin, HJV, hemojuvelin; HJVz1, hemojuvelin zone 1; HJVz2, hemojuvelin zone 2; RBC, red blood cell count; sTfR, soluble transferrin receptor; TfR2, transferrin receptor 2; TfR2z1, transferrin receptor 2 zone 1; TfR2z2, transferrin receptor 2 zone 2; TSAT, transferrin saturation; UIBC, unsaturated iron binding capacity.

∗ *P* < 0.050.

∗∗ *P* < 0.010.

∗∗∗ *P* < 0.001.

Linear correlation analysis between inflammatory markers and percentage DNA methylation of each CpG site is shown in Supplementary Table 5. CRP negatively correlated with *TfR2*z1 CpG 5 (*r =* –0.316, *P <* 0.050), CpG 6 (*r =* –0.469, *P <* 0.001), CpG 8 (*r =* –0.350, *P <* 0.010), CpG 10 ( *r =* –0.428, *P <* 0.001), and mean zone methylation *r =* –0.344, *P <* 0.010); and with *TfR2z2* CpG 2 (*r =* 0.276, *P <* 0.050), CpG 5 (*r =* –0.334, *P <* 0.010), CpG 6 (*r =* –0.265, *P <* 0.050) (Supplemental Table 5). IFN-γ was not associated with DNA methylation at any of the CpG sites. TNF-α was positively associated with DNA methylation at *TfR2*z1 CpG 1 (*r =* 0.353, *P <* 0.001), CpG 2 (*r =* 0.274, *P <* 0.050), *TfR2*z2 CpG 1 (*r =* 0.422, *P <* 0.001). IL-6 showed negative correlation with *TfR2*z1 CpG 5 (*r =* –0.282, *P <* 0.050), and mean zone methylation *r =* –0.315, *P <* 0.050); and positive with *HJV*z1 CpG 5 (*r =* 0.263, *P <* 0.050). IL-1β positively correlated with DNA methylation at *HJV*z2 CpG 1 (*r =* 0.333, *P <* 0.010). The mean percentage methylation of both zones of *TfR2* correlated negatively with CRP (*r =* –0.329, *P <* 0.01) and IL-6 (*r =* –0.304, *P <* 0.05). However, the mean percentage methylation of both zones of *HJV* did not correlate with any inflammatory markers.

## Discussion

This study examined the association between maternal adiposity and DNA methylation of *TfR2* and *HJV* genes at 12 GW, as well as their relationship with iron and inflammatory markers. Women with obesity showed lower DNA methylation at *TfR2z1* and higher methylation at *HJVz2* compared to normal-weight women. Additionally, greater inflammation in the context of increased adiposity was linked to reduced *HJVz1* methylation.

High BMI and central adiposity are linked to lower DNA methylation in two regions that may regulate *TfR2* expression. This has not been previously reported in humans, although animal studies showed similar trends [45,46]. Despite some inconsistencies [47,48], several studies report higher hemoglobin and/or RBC concentrations in pregnant women with obesity compared to those of normal weight [7,10,49]. In our analysis, RBC, hemoglobin, and CRP concentrations, and adiposity, were negatively correlated with *TfR2* DNA methylation indicating that increased RBC and hemoglobin concentrations may relate to higher *TfR2* expression. However, the association between adiposity and *TfR2* DNA methylation remained independent of hemoglobin concentrations. Although CRP may contribute to this epigenomic change, the lack of association between other inflammatory markers and *TfR2* methylation suggests that additional mechanisms may be involved, such as oxidative stress and reactive oxygen species [50].

Lower methylation of the *TfR2* gene in women with obesity may increase its expression, promoting higher hepatic hepcidin production and influencing erythropoiesis through its interaction with the erythropoietin receptor (EpoR) complex. TfR2 is essential for EpoR transport to the cell surface and supports terminal differentiation of erythroid progenitors, particularly under iron-deficient conditions [51]. In the context of obesity, elevated *TfR2* expression could mimic an iron-replete state, reducing EPO sensitivity and impairing erythropoiesis. This dysregulation may result in a failure to meet the heightened iron demands of pregnancy, leaving women with compromised iron metabolism.

*HJV* may be negatively regulated in obesity, reducing hepcidin production in early pregnancy and reflecting impaired iron status. HJV originates mainly from the liver, where it regulates hepcidin in response to iron signals, and from skeletal muscle as soluble hemojuvelin, which may suppress hepcidin under ID or hypoxia [52,53]. In this analysis, women with higher BMI and central adiposity showed increased *HJV* methylation, independent of hemoglobin, suggesting reduced hepatic hepcidin. Hepcidin was not correlated with *HJV* methylation, whereas serum iron, TSAT, and UIBC were, implying that iron regulation involves mechanisms beyond the hepcidin pathway. These findings may reflect a compensatory response to low iron concentrations in the context of heightened iron demand, such as during pregnancy.

This study found that inflammation mediates the association between adiposity and *HJV* methylation. In women with both high adiposity and inflammation, *HJV* methylation was significantly reduced, an effect not seen with either condition alone. This decrease in *HJV* methylation might lead to an increased HJV expression, which could mimic a state of iron overload. To our knowledge, this is the first study to evaluate the association between proinflammatory cytokines and *HJV* methylation in human blood. Our findings align with Luciani et al. [54] who observed elevated *HJV* expression in adipose tissue of women with morbid obesity. Furthermore, our findings are consistent with previous evidence showing that both HJV and hepcidin are upregulated at the transcriptional level in mice during inflammation [53]. Under physiological conditions, HJV expression is regulated by iron status; however, inflammation may override this control, inducing sustained hepcidin activation and reducing iron availability independently of systemic iron concentrations [26,53]. IL-6 and IL-1β are known to promote hepcidin synthesis through transcriptional regulation; however, the role of DNA methylation in regulating *HJV* expression under inflammation and obesity has not been previously investigated [31,55]. Beyond the individual correlations between CpG 5 of *HJVz1* and IL-6, and CpG 1 of *HJVz2* and IL-1β (Supplementary Table 5), no significant associations were observed with other inflammatory markers. These findings suggest that elevated IL-6, IL-1β, and possibly additional unmeasured mediators may contribute to reduced *HJV* methylation, potentially increasing *HJV* expression. Regardless of iron status, this would promote lower iron availability by increasing hepcidin expression in early pregnancy.

The current study highlights a potential interplay between inflammation and high adiposity and differential methylation of the *HJV* gene, representing a novel, previously undescribed regulatory mechanism. This may help explain why elevated hepcidin concentrations have only been observed in some pregnant women with obesity [8]. In our study, hepcidin concentrations did not differ between BMI groups, suggesting that hepcidin increases only in a subset of pregnant women when chronic inflammation and adequate iron stores coexist, where inflammatory signaling plays a dominant role. Furthermore, chronic inflammation likely affects only a subgroup of women with obesity, which could explain the variability of hepcidin findings across studies. In the presence of chronic inflammation, hepcidin appears to be less sensitive to iron status, contributing to impaired iron homeostasis. Consistent with this complex regulation, in our recently published study, iron supplementation with 17 mg of elemental iron per day during pregnancy, administered uniformly to all participants, did not result in differences in the incidence of anemia across BMI groups. However, despite this supplementation, the prevalence of ID at 36 GW remained extremely high (40%). Importantly, women who presented with both obesity and elevated inflammation in the first trimester had a higher risk of ID at 36 GW, whereas this association was not observed when comparing obesity with normal weight or overweight alone [15]. These findings further support the hypothesis that inflammation plays a mechanistic role in iron metabolism.

This study has limitations that should be acknowledged. The cross-sectional observational design restricts inference of causal relationships, and the absence of an overweight group may limit comparability. Additionally, DNA methylation analysis was conducted using white blood cells rather than liver tissue, which would have provided more specific insights into hepcidin synthesis. Although ethnic data were unavailable, the cohort was predominantly white (>99%). Single cytokine measurements cannot distinguish chronic from transient inflammation, and it remains unclear whether methylation changes correspond to gene expression, requiring confirmation by mRNA analysis.

Despite these limitations, this study has notable strengths. It is the first to compare *HJV* and *TfR2* DNA methylation in pregnancy between women of normal weight and obesity during the first trimester. A comprehensive range of hematologic, iron, and inflammatory markers was assessed. Furthermore, adiposity was evaluated using multiple measures beyond BMI, providing a more detailed assessment of body composition.

In conclusion, this study has shown that increased adiposity in the first trimester was associated with reduced *TfR2* and increased *HJV* DNA methylation, independent of hemoglobin, iron status and inflammation. Although inflammation alone had no effect, its coexistence with obesity was linked to decreased *HJV* methylation. Given the higher prevalence of inflammation among women with obesity, this subgroup may experience hepcidin dysregulation, increasing the risk of ID and anemia during pregnancy.

## Author contributions

The authors’ responsibilities were as follows – SPD, REI, PDT, MSM, MAK, ML, MTM: designed research; SPD, KLR, CC, EPR: conducted research; SPD: analyzed data; SPD, REI, MTM: wrote the paper; MTM: had primary responsibility for final content; and all authors: read and approved the final manuscript.

## Data availability

Data described in the manuscript, code book, and analytic code will be made available upon request.

## Funding

SPD is a PhD Researcher funded by the Department for the Economy scholarship, Ulster University. This research received funding from Solvotrin Therapeutics.

## Conflict of interest

MTM reports financial support was provided by Solvotrin Therapeutics. MAK reports financial support was provided by Solvotrin Therapeutics. MSM reports financial support was provided by Solvotrin Therapeutics. MTM reports a relationship with Solvotrin Therapeutics that includes: funding grants. MAK reports a relationship with Solvotrin Therapeutics that includes: funding grants. MSM reports a relationship with Solvotrin Therapeutics that includes: funding grants. ML has patent #WO2017158030A1. Compositions and methods for increasing iron intake in a mammal issued to Trinity College Dublin and Solvotrin Therapeutics. All other authors report no conflicts of interest.
