# Supplementary material for: Maternal Adiposity and DNA Methylation of TfR2 and HJV Genes in Early Pregnancy: Mediating Role of Inflammation and Consequences for Iron Status
Source: J Nutr. 2026 Feb 4;156(4):101393. doi: 10.1016/j.tjnut.2026.101393 (PMC13084681; doi:10.1016/j.tjnut.2026.101393)
Supplement: multimedia component 1 [file mmc1.docx]

**Supplementary tables**

**Supplementary table 1** Characteristics of the primers selected for DNA methylation analysis within the *TfR2* gene.

A)

| ***TfR2 zone 1 (TfR2z1)*** | |
| --- | --- |
| *Sequence location* | chr7:100,633,629-100,633,749 |
| *CpG island* | CpG island 57 |
| *Promoter/Enhancer area involved* | Enhancer |
| *CpG locations* | CpG 1: 100,633,629, CpG 2: 100,633,637, CpG 3: 100,633,652, CpG 4: 100,633,655, CpG 5: 100,633,683, CpG 6: 100,633,690, CpG 7: 100,633,692, CpG 8: 100,633,696, CpG 9: 100,633,731, CpG 10: 100,633,739, and CpG 11: 100,633,747 |
| *Forward primer sequence* | GGGAAGAGGGAAGGATGTTA |
| *Reverse primer sequence* | AATCCACTACACTCCAACCTAA |
| *Sequencing primer* | GGGAAGGATGTTAGAGA |
| *Sequence to BLAT* | CGTGGGGTCGCCAGGGGCAGGGGCGGCGAGGGGTTCCTGAGTTTAG  GAAAAGAGCGCTCCCCGCGGACGCTTTTTTTTTTTTTTTTTTTTTTTTTTTT  GAGACGGAGTGTCGCTCTGTCGC |
| *Annealing temperature pyro-PCR* | 55.5 °C |

B)

| ***TfR2 zone 2 (TfR2z2)*** |  |
| --- | --- |
| *Sequence location* | chr7:100,641,125-100,641,259 |
| *CpG island* | No |
| *Promoter/Enhancer area involved* | Enhancer |
| *CpG locations* | CpG 1: 100,641,127, CpG 2: 100,641,141, CpG 3: 100,641,173, CpG 4: 100,641,188, CpG 5: 100,641,198, CpG 6: 100,641,238 |
| *Forward primer sequence* | TTATGGGGTAGAAGTGGGTTAAT |
| *Reverse primer sequence* | ACCCCCCAAACCTCTTAC |
| *Sequencing primer* | AGAAGTGGGTTAATGTTTCCGCCCCCTCCTCCCCGTCTTCCTCTTCCTCCTCCAGGTGCCCTTTCC |
| *Sequence to BLAT* | GGGGGCCTTCCACACGCTGGTAGACGGTCTGAGAGGATCTTGGGGAC AGTTGTTGCTGTGCAGGCGAGGTGGGCATGAGATTGGGG |
| *Annealing temperature pyro-PCR* | 56.3 °C |

A) *TfR2* zone 1; B) *TfR2* zone 2. CpG, 5'—C—phosphate—G—3'; BLAT, BLAST-Like alignment tool; TfR2, transferrin receptor 2; PCR, Polymerase Chain Reaction.

**Supplementary table 2** Characteristics of the primers selected for DNA methylation analysis within the *HJV* gene.

A)

| ***HJV zone 1 (HJVz1)*** | |
| --- | --- |
| Sequence location | chr1:146,019,296-146,019,383 |
| *CpG island* | CpG island 29 |
| *Promoter/Enhancer area involved* | Enhancer |
| CpG locations | CpG 1: 146,019,305, CpG 2: 146,019,321, CpG 3: 146,019,330, CpG 4: 146,019,346, CpG 5: 146,019,353, CpG 6: 146,019,365, CpG 7: 146,019,374 |
| *Forward primer sequence* | TTTGGATAAGGTATGTGTGAAAGTGAT |
| *Reverse primer sequence* | CCCTACCCCCAACCCTTATAACTA |
| *Sequencing primer* | GTGTGAAAGTGATGG |
| *Sequence to BLAT* | TGGAAGCTGCGCACATGGGGGTCCCCGAAGGAAGCGCAATGCAAGAA  CCCCGGGGGACGACCATGCAGCCGGGAAAACCGGCCTTCAT |
| *Annealing temperature pyro-PCR* | 53 °C |

B)

| ***HJV zone 2 (HJVz2)*** | |
| --- | --- |
| *Sequence location* | chr1:146,021,811-146,021,912 |
| *CpG island* | No |
| *Promoter/Enhancer area involved* | Promoter |
| *CpG locations* | CpG 1: 146,021,819, CpG 2: 146,021,855. |
| *Forward primer sequence* | GGTTGGTTAGAGTATTGGATAGTT |
| *Reverse primer sequence* | CTAACTTAACACTCAATACTCCATATTCT |
| *Sequencing primer* | GAGGGAGGTGGTTAT |
| *Sequence to BLAT* | TGAGTTGACGGTTTTAAAAATAGCTAAGTCCAAAATT CCAGGAACGTTGGTGGGGGTAGGGTGTGGGAGTTGG  GGGAGTGGGGAGGGTTAGAGGATTAGAAA |
| *Annealing temperature pyro-PCR* | 56 °C |

1. *HJV* zone 1; B) *HJV* zone 2. CpG, 5'—C—phosphate—G—3'; HJV, hemojuvelin gene. BLAT, BLAST-Like alignment tool; PCR, Polymerase Chain Reaction.

**Supplementary Table 3** Concentrations of iron and pro-inflammatory markers in pregnant women with normal weight and obesity.

|  | **Normal weight** (n=34) | | **Obesity** (n=31) | |  |
| --- | --- | --- | --- | --- | --- |
|  | *Mean* | *SD* | *Mean* | *SD* | *p-value* |
| Hemoglobin (g/L) | 126.5 | 8.2 | 132.0 | 8.6 | **0.017** |
| Ferritin (µg/L) | 51.1 | 33.7 | 59.2 | 49.8 | 0.694 |
| Serum iron (µmol/L) | 19.6 | 4.5 | 15.7 | 6.2 | **0.009** |
| TSAT (%) | 29.9 | 8.6 | 23.8 | 10.5 | **0.011** |
| TIBC (µmol/L) | 67.0 | 9.3 | 68.4 | 10.1 | 0.577 |
| UIBC (µmol/L) | 47.5 | 10.5 | 52.5 | 12.9 | 0.072 |
| sTfR(µg/mL) | 0.942 | 0.54 | 1.147 | 0.59 | 0.172 |
| Hepcidin (pg/mL)* | 11144 | 5100, 22077 | 12393 | 5979, 22656 | 0.173 |
| CRP (mg/L) | 4.44 | 5.18 | 11.7 | 10.8 | **<0.001** |
| TNF-α, (fg/mL) | 691.0 | 188 | 725.1 | 145 | 0.287 |
| IFN-γ (fg/mL) | 541.1 | 273 | 530.8 | 218 | 0.875 |
| IL-6 (fg/mL) | 1423.2 | 643 | 2231.8 | 1381 | **0.002** |
| IL-1β (fg/mL) | 139.9 | 96.4 | 167.1 | 93.9 | 0.218 |

Data presented as mean, SD. *Reported in median and interquartile range. n=65. General linear model adjusted for maternal age, smoking, educational status. CRP, C-reactive protein; IFN, interferon; IL, interleukin; IQR, interquartile range; sTfR, soluble transferrin receptor; TIBC, total iron binding capacity; TNF, Tumor necrosis factor; TSAT, transferrin saturation; UIBC, unsaturated iron binding capacity.

**Supplementary table** **4A** Mean DNA methylation percentage at each CpG site, as well as the mean methylation percentage across all CpG sites within each analyzed region of the *TfR2* and *HJV* genes, according to CRP concentrations.

|  | **CRP≤5 mg/L** (n=35) | | **CRP>5 mg/L (**n=30) | |  |
| --- | --- | --- | --- | --- | --- |
|  | *Mean* | *SD* | *Mean* | *SD* | *p-value* |
| ***TFR2*z1 (%)** |  |  |  |  |  |
| CpG 1 | 3.82 | 1.1 | 3.65 | 1.0 | 0.717 |
| CpG 2 | 50.3 | 1.8 | 4.84 | 1.7 | 0.996 |
| CpG 3 | 5.81 | 1.1 | 5.46 | 1.4 | 0.493 |
| CpG 4 | 4.39 | 1.0 | 4.07 | 1.0 | 0.360 |
| CpG 5 | 7.78 | 1.5 | 6.69 | 1.4 | **0.012** |
| CpG 6 | 12.5 | 2.3 | 9.80 | 2.2 | **<0.001** |
| CpG 7 | 4.57 | 0.9 | 4.57 | 1.2 | 0.795 |
| CpG 8 | 6.00 | 2.1 | 5.04 | 1.4 | 0.073 |
| CpG 9 | 6.75 | 2.0 | 6.15 | 1.4 | 0.224 |
| CpG 10 | 12.3 | 2.3 | 10.0 | 2.0 | **<0.001** |
| CpG 11 | 6.03 | 1.3 | 5.84 | 1.7 | 0.973 |
| Mean of all CpG sites | 6.82 | 1.2 | 6.01 | 1.1 | **0.030** |
|  |  |  |  |  |  |
| ***TFR2*z2 (%)** |  |  |  |  |  |
| CpG 1 | 16.0 | 4.5 | 14.1 | 3.8 | 0.148 |
| CpG 2 | 13.4 | 2.8 | 14.5 | 2.0 | 0.203 |
| CpG 3 | 26.1 | 5.2 | 28.4 | 5.8 | 0.159 |
| CpG 4 | 12.2 | 6.9 | 9.38 | 1.4 | 0.085 |
| CpG 5 | 15.2 | 4.6 | 11.9 | 1.8 | **0.001** |
| CpG 6 | 20.5 | 5.6 | 17.2 | 2.4 | **0.008** |
| Mean of all CpG sites | 17.2 | 2.8 | 15.9 | 1.8 | **0.029** |
|  |  |  |  |  |  |
| ***HJ*Vz1 (%)** |  |  |  |  |  |
| CpG 1 | 49.1 | 7.1 | 50.6 | 4.1 | 0.277 |
| CpG 2 | 51.0 | 7.6 | 52.6 | 10.5 | 0.511 |
| CpG 3 | 43.6 | 5.2 | 45.2 | 4.4 | 0.271 |
| CpG 4 | 26.1 | 7.0 | 26.2 | 9.9 | 0.960 |
| CpG 5 | 13.2 | 4.0 | 13.2 | 4.6 | 0.960 |
| CpG 6 | 17.2 | 6.9 | 16.9 | 6.9 | 0.785 |
| CpG 7 | 15.2 | 7.4 | 15.9 | 8.5 | 0.813 |
| Mean of all CpG sites | 30.8 | 4.9 | 31.5 | 5.8 | 0.629 |
|  |  |  |  |  |  |
| ***HJV*z2 (%)** |  |  |  |  |  |
| CpG 1 | 85.6 | 5.5 | 86.7 | 2.0 | 0.388 |
| CpG 2 | 39.7 | 6.0 | 41.5 | 5.7 | 0.302 |
| Mean of all CpG sites | 62.7 | 4.4 | 64.1 | 3.3 | 0.225 |
|  |  |  |  |  |  |

Data presented as mean ± SD. "Mean of all CpG sites" refers to the mean percentage of DNA methylation across all CpG sites within the zone being assessed. General linear model adjusted for maternal age, smoking, educational status and hemoglobin at 12 GW.

CpG, cytosine and guanine separated by a single phosphate group; CRP, C-reactive protein; HJV, hemojuvelin; HJVz1, hemojuvelin zone 1; HJVz2, hemojuvelin zone 2; TfR2, transferrin receptor 2; TfR2z1, Transferrin receptor 2 zone 1; TfR2z2, transferrin receptor zone 2.

**Supplementary table 4B** Mean DNA methylation percentage at each CpG site, as well as the mean methylation percentage for each analyzed zone of the *TfR2* and *HJV* genes, according to the inflammation score.

|  | **Low inflammation score** (n=22) | | **High inflammation score** (n=43) | |  |
| --- | --- | --- | --- | --- | --- |
|  | *Mean* | *SD* | *Mean* | *SD* | *p-value* |
| **TFR2z1 (%)** |  |  |  |  |  |
| CpG 1 | 3.50 | 1.01 | 3.79 | 1.06 | 0.230 |
| CpG 2 | 4.68 | 1.83 | 5.04 | 1.64 | 0.543 |
| CpG 3 | 5.63 | 1.21 | 5.62 | 1.36 | 0.856 |
| CpG 4 | 4.22 | 0.97 | 4.21 | 1.06 | 0.976 |
| CpG 5 | 7.31 | 1.25 | 7.11 | 1.73 | 0.814 |
| CpG 6 | 11.2 | 2.58 | 11.1 | 2.63 | 0.814 |
| CpG 7 | 4.41 | 0.85 | 4.62 | 1.19 | 0.508 |
| CpG 8 | 5.45 | 1.01 | 5.51 | 2.17 | 0.832 |
| CpG 9 | 6.41 | 1.05 | 6.37 | 2.09 | 0.871 |
| CpG 10 | 11.5 | 2.01 | 11.0 | 2.62 | 0.447 |
| CpG 11 | 5.91 | 0.97 | 5.83 | 1.73 | 0.827 |
| Mean of all CpG sites | 6.39 | 1.00 | 6.39 | 1.38 | 0.980 |
|  |  |  |  |  |  |
| **TFR2z2 (%)** |  |  |  |  |  |
| CpG 1 | 13.8 | 3.96 | 16.2 | 4.20 | 0.092 |
| CpG 2 | 14.4 | 2.61 | 14.1 | 2.79 | 0.455 |
| CpG 3 | 27.2 | 5.27 | 27.3 | 5.41 | 0.918 |
| CpG 4 | 12.2 | 8.48 | 10.2 | 1.94 | 0.247 |
| CpG 5 | 13.9 | 3.06 | 13.5 | 4.21 | 0.913 |
| CpG 6 | 18.7 | 4.20 | 19.2 | 4.83 | 0.694 |
| Mean of all CpG sites | 16.7 | 2.45 | 16.7 | 2.43 | 0.939 |
|  |  |  |  |  |  |
| **HJVz1 (%)** |  |  |  |  |  |
| CpG 1 | 49.8 | 4.38 | 45.9 | 6.38 | 0.809 |
| CpG 2 | 52.9 | 9.0 | 51.2 | 8.64 | 0.518 |
| CpG 3 | 44.3 | 4.44 | 44.3 | 5.15 | 0.929 |
| CpG 4 | 25.9 | 8.28 | 27.2 | 8.70 | 0.826 |
| CpG 5 | 12.7 | 4.47 | 14.1 | 4.49 | 0.479 |
| CpG 6 | 16.1 | 6.64 | 18.4 | 7.31 | 0.371 |
| CpG 7 | 15.1 | 8.02 | 16.6 | 8.04 | 0.650 |
| Mean of all CpG sites | 31.0 | 5.26 | 31.6 | 5.45 | 0.840 |
|  |  |  |  |  |  |
| **HJVz2 (%)** |  |  |  |  |  |
| CpG 1 | 86.1 | 5.15 | 85.9 | 3.93 | 0.912 |
| CpG 2 | 39.8 | 5.47 | 40.7 | 5.88 | 0.308 |
| Mean of all CpG sites | 63.0 | 4.05 | 63.4 | 3.98 | 0.435 |
|  |  |  |  |  |  |

Data presented as mean ± SD. "Mean of all CpG sites" refers to the mean percentage of DNA methylation across all CpG sites within the zone being assessed. Low inflammation score (<8), n=22, high inflammation score (≥8), n=43. General linear model adjusted for maternal age, smoking, educational status and hemoglobin at 12GW.

CpG, cytosine and guanine separated by a single phosphate group; HJV, hemojuvelin; HJVz1, hemojuvelin zone 1; HJVz2, hemojuvelin zone 2; TfR2, transferrin receptor 2; TfR2z1, Transferrin receptor 2 zone 1; TfR2z2, transferrin receptor zone 2.

**Supplementary table 5** Relationship between inflammatory markers concentrations and percentage DNA methylation of *TfR2* and *HJV.*

|  | **CRP mg/L** | **IFN-γ fg/mL** | **TNF-α fg/mL** | **IL-6 fg/mL)** | **IL-1β fg/mL** |
| --- | --- | --- | --- | --- | --- |
| ***TFR2*z1 (%)** |  |  |  |  |  |
| CpG 1 | -0.138 | -0.111 | **0.353***** | 0.011 | 0.11 |
| CpG 2 | -0.123 | -0.099 | **0.274*** | 0.128 | 0.181 |
| CpG 3 | -0.165 | 0.194 | 0.030 | 0.039 | -0.146 |
| CpG 4 | -0.154 | 0.038 | 0.164 | -0.028 | -0.072 |
| CpG 5 | **-0.316*** | -0.028 | -0.016 | -0.176 | -0.196 |
| CpG 6 | **-0.469***** | -0.026 | 0.077 | -0.186 | -0.084 |
| CpG 7 | -0.056 | 0.058 | 0.156 | 0.141 | -0.129 |
| CpG 8 | -**0.350**** | 0.080 | 0.088 | -0.166 | -0.169 |
| CpG 9 | -0.213 | 0.018 | 0.016 | -0.123 | -0.224 |
| CpG 10 | **-0.428***** | -0.023 | 0.023 | -0.241 | -0.136 |
| CpG 11 | -0.124 | 0.001 | 0.074 | -0.136 | -0.061 |
| Mean of all CpG sites | **-0.344**** | 0.006 | 0.126 | -0.121 | -0.119 |
|  |  |  |  |  |  |
| ***TFR2*z2 (%)** |  |  |  |  |  |
| CpG 1 | -0.168 | 0.009 | **0.422***** | -0.126 | 0.159 |
| CpG 2 | **0.276*** | -0.004 | 0.158 | -0.073 | -0.095 |
| CpG 3 | 0.198 | -0.125 | 0.050 | -0.111 | 0.074 |
| CpG 4 | -0.111 | 0.158 | -0.206 | -0.222 | -0.132 |
| CpG 5 | **-0.334**** | 0.241 | 0.100 | **-0.282*** | -0.118 |
| CpG 6 | **-0.265*** | 0.238 | 0.098 | -0.220 | -0.191 |
| Mean of all CpG sites | -0.137 | 0.152 | 0.155 | **-0.315*** | -0.083 |
|  |  |  |  |  |  |
| ***HJV*z1 (%)** |  |  |  |  |  |
| CpG 1 | 0.051 | 0.051 | -0.085 | -0.019 | 0.198 |
| CpG 2 | 0.221 | 0.027 | -0.095 | 0.137 | -0.050 |
| CpG 3 | 0.223 | -0.020 | -0.094 | 0.153 | 0.063 |
| CpG 4 | 0.138 | 0.027 | -0.105 | 0.232 | -0.080 |
| CpG 5 | 0.141 | -0.004 | 0.071 | **0.263*** | -0.039 |
| CpG 6 | 0.148 | 0.015 | 0.039 | 0.237 | -0.021 |
| CpG 7 | 0.154 | 0.045 | -0.048 | 0.228 | -0.030 |
| Mean of all CpG sites | 0.200 | 0.030 | -0.066 | 0.227 | -0.006 |
|  |  |  |  |  |  |
| ***HJV*z2 (%)** |  |  |  |  |  |
| CpG 1 | -0.018 | -0.098 | -0.007 | -0.017 | **0.333**** |
| CpG 2 | 0.129 | 0.019 | 0.042 | -0.025 | 0.007 |
| Mean of all CpG sites | 0.083 | -0.040 | 0.026 | -0.027 | 0.187 |

Pearson linear correlation. "Mean of all CpG sites" refers to the mean percentage of DNA methylation across all CpG sites within the zone being assessed. Data presented as coefficient r. * p<.050, ** p<0.010, *** p<0.001. CpG, cytosine and guanine separated by a single phosphate group; CRP, C-reactive protein; HJV, hemojuvelin; HJVz1, hemojuvelin zone 1; HJVz2, hemojuvelin zone 2; IFN, interferon; IL, interleukin; TNF, Tumor necrosis factor; TfR2, transferrin receptor 2; TfR2z1, Transferrin receptor 2 zone 1; TfR2z2, transferrin receptor zone 2.
